# Oxidative Status and Lipid Metabolism Analytes in Dogs with Mast Cell Tumors: A Preliminary Study

**DOI:** 10.3390/antiox13121473

**Published:** 2024-11-29

**Authors:** Argyrios Ginoudis, Dimitra Pardali, Mathios E. Mylonakis, Androniki Tamvakis, Asta Tvarijonaviciute, Evgenia Lymperaki, Jose Joaquin Ceron, Zoe Polizopoulou

**Affiliations:** 1 Diagnostic Laboratory, School of Veterinary Medicine, Faculty of Health Sciences, Aristotle University of Thessaloniki, 546 27 Thessaloniki, Greece; agkinou@vet.auth.gr (A.G.); dpardali@vet.auth.gr (D.P.); 2 Companion Animal Clinic, School of Veterinary Medicine, Faculty of Health Sciences, Aristotle University of Thessaloniki, 546 27 Thessaloniki, Greece; mmylonak@vet.auth.gr; 3 Laboratory of Ecology and System Dynamics, Department of Marine Sciences, University of the Aegean, 811 00 Mytilene, Greece; atamvaki@mar.aegean.gr; 4 Interdisciplinary Laboratory of Clinical Analysis, Interlab-UMU, Regional Campus of International Excellence Campus Mare Nostrum, University of Murcia, 30100 Murcia, Spain; asta@um.es (A.T.); jjceron@um.es (J.J.C.); 5 Department of Biomedical Sciences, International Hellenic University, 570 22 Sindos, Greece; evlimper@ihu.gr

**Keywords:** canine, mast cell tumors, Paraoxonase-1 (PON-1), metabolomics, redox status, biomarkers

## Abstract

Mast cell tumors (MCTs) are common skin neoplasms in dogs. Prognostic indicators include histologic grade, clinical stage, high Ki-67 index, elevated argyrophilic nucleolus organizer regions (AgNOR) index, *c-kit* mutations, and recurrence after surgery. Blood serum redox status has been shown to correlate with prognostic factors in canine lymphoma and mammary tumors. This study aimed to assess the correlation between established prognostic factors and serum redox status and lipid metabolism analytes in dogs with MCTs. Dogs with cutaneous (*n* = 33) or subcutaneous (*n* = 6) MCTs, without comorbidities, were studied. Staging was evaluated based on cytology of regional lymph nodes and ultrasound-guided liver and spleen aspiration cytology. Histologic grading and immunohistochemical staining for Ki-67 and KIT patterns were performed on excised tumor specimens. Dogs were categorized by Patnaik grading (1–3), Kiupel grading (low/high), metastatic status, Ki-67 positive nuclei per cm^2^ (>23 or ≤23), and KIT pattern (I, II–III). Paraoxonase-1, Butyrylcholinesterase, Cupric Reducing Antioxidant Capacity (CUPRAC), Diacron Reactive Oxygen Metabolites (d-ROMs), and oxy-adsorbent levels were measured before any therapeutic intervention. ANOVA and independent *t*-tests were used to detect differences in the mean values among groups. Paraoxonase-1 activity was significantly lower in Patnaik grade 3 (*p* = 0.003) and Kiupel high-grade (*p* = 0.022) MCTs. No significant differences were found in CUPRAC, d-ROMs, or oxy-adsorbent levels across different prognostic groups. This study found a significant correlation between histologic grading and Paraoxonase-1 activity, suggesting a potential role of Paraoxonase-1 as a prognostic biomarker in canine MCTs.

## 1. Introduction

Mast cell tumors (MCTs) are the most common malignant skin neoplasm in dogs with an incidence rate of 16–21% [1]. The biological behavior of canine MCTs varies considerably, ranging from benign tumors amenable to curative surgery, to extremely aggressive tumors, with a survival time of less than four months, regardless of the treatment approach [2]. Subcutaneous MCTs tend to have a more favorable prognosis compared to cutaneous MCTs in dogs [3,4].

Historically, the most important prognostic markers in dogs with MCT included histologic grading (based mostly on a 2-tier system) and the clinical stage of the neoplasm [2,5]. In addition to this, molecular markers, including c-kit expression pattern, *c-kit* mutations and proliferation indices such as the Ki-67 index, which correlates with the growth fraction of the tumor, and the argyrophilic nucleolus organizer regions (AgNORs) index, which correlates with the proliferation rate of the neoplastic cells, are of particular prognostic importance [6,7,8,9,10,11].

Redox status is defined as the dynamic balance between oxidants and antioxidants to maintain cell and tissue function [12]. Disruption of this balance triggers oxidative stress, which has been linked to the pathogenesis of many diseases such as cancer, cardiovascular disease, and diabetes mellitus in humans [13]. The total oxidative status of blood can be assessed by direct measurement of Reactive Oxygen Species (ROS), with methods assessing the oxidation of ROS-sensitive molecules [14,15,16] or by determining the concentration of molecules derived from oxidation of biomolecules, including proteins, lipids, and nucleic acids using various assays [17]. Oxidative stress can be estimated indirectly by measuring enzymes that induce the body’s antioxidant response (Thioredoxin, Peroxiredoxins), high concentrations of which are indicative of oxidative stress [18]. Finally, oxidative stress is assessed by determining the concentration of antioxidants such as ascorbic acid, glutathione, paraoxonase 1 (PON-1) and vitamin E compounds (α-, γ-tocopherol) [19,20,21]. Determination of total antioxidants can be performed by estimating the oxidant reducing capacity contained in commercially available reagents [22].

PON-1 in particular is an important enzyme involved in lipid metabolism and has gained attention as biomarker of oxidative stress and disease. Primarily associated with high-density lipoproteins (HDL), PON-1 plays a key role in protecting lipids from oxidative damage by hydrolyzing lipid peroxides, thus maintaining the antioxidant capacity of HDL and preventing atherosclerosis [23,24]. Its activity is decreased in various pathological conditions, including cancer, where oxidative stress plays a pivotal role in disease progression [25]. Butyrylcholinesterase (BChE), although primarily recognized for its role in hydrolyzing choline-based esters, is increasingly implicated in lipid metabolism and inflammation. Elevated or reduced BChE levels have been associated with metabolic disorders, chronic inflammation, and cancer, making it a potential biomarker for disease states [26,27,28].

Several studies have reported on the measurement of blood redox status in human cancers. For instance, oxidative stress measured by 8-hydroxydeoxyguanosine (8-OHdG) has been linked to cancer risk, while elevated Malondialdehyde (MDA) and reduced vitamin E levels are associated with poor breast cancer prognosis [29,30]. Similarly, higher 8-OHdG concentrations have been linked to worse outcomes in ovarian carcinoma [31].

In canine medicine, blood redox status has been used to monitor treatment outcomes for leishmaniosis. A study showed significant increases in antioxidant markers [Cupric Reducing Antioxidant Capacity (CUPRAC), serum thiol, PON-1] after 30 and 180 days of treatment [32]. Redox status has also been evaluated in canine inflammatory bowel disease (IBD), where oxidative markers were higher and antioxidant markers lower in affected dogs, suggesting oxidative stress plays a role in IBD pathogenesis [33]. However, no correlation between these markers and treatment success was assessed.

In a study that assessed oxidative damage in dogs with multicentric lymphoma or with cutaneous MCT, serum levels of O^2−^, and NO appeared reduced in diseased compared to clinically healthy dogs, with the antioxidant response showing no statistically significant difference [34]. In another study including dogs with MCT, the oxidative status of the diseased dogs was assessed by measuring d-ROMs, which were found to be increased compared to a healthy dog population, while antioxidant capacity (assessed by the BAP method) was found to be reduced in the diseased animals, providing a basis for further study [35]. In the above studies, the potential prognostic significance of oxidative stress was not addressed, particularly in relation to well-established prognostic markers such as the clinical stage and histologic grade of the disease.

The objectives of this study were to assess the blood redox status and lipid metabolism markers in dogs with MCTs and to investigate their possible correlation with the clinical stage, the histologic grade, and the immunohistochemical assessment of the disease.

## 2. Materials and Methods

### 2.1. Study Population

Adult dogs with confirmed cutaneous or subcutaneous MCTs based on cytologic examination were included in the study. The patients were required to have not undergone surgical or chemotherapy treatment for the MCTs prior to inclusion in the study, and to have no comorbidities that might affect the redox status of the dogs.

### 2.2. History, Physical Examination, and Clinical Staging

In all cases, a detailed history was obtained, followed by a physical examination with particular attention being paid to the presence of any cutaneous and subcutaneous masses, or any manifestations implying comorbidity. The anatomic location of the mass(es) was mapped and its size was measured with a thickness gauge. All masses and palpable lymph nodes were fine needle aspirated. Diagnostic imaging included abdominal ultrasound followed by fine needle aspiration of the liver and spleen according to the methodology for staging [36,37]. All aspirates were Giemsa-stained and examined for the presence of mast cells. Metastasis was diagnosed by the presence of atypical mast cells and/or clusters of normal-looking mast cells in the lymph nodes, the liver, and the spleen.

### 2.3. Clinicopathologic Testing

All the dogs included in the study underwent a complete blood count (CBC) (ADVIA 2120 Hematology System, Siemens Healthcare Diagnostics, Tarrytown, NY, USA) and serum biochemistry panel (Vitalab Flexor E Chemistry Analyzer, Vital Scientific N.V., Dieren, The Netherlands) to exclude any underlying conditions that could influence the biomarkers being studied. These tests ensured that no severe comorbidities were present, allowing for a more accurate assessment of the relationship between the biomarkers and mast cell tumors (MCTs) without interference from other health issues.

### 2.4. Histologic and Immunohistochemical Examination

After surgical excision of the primary neoplasms and/or any lymph nodes with cytologic evidence of metastasis, the excised tissues were sent for histologic examination in order to confirm the diagnosis, definitively characterize the MCT as cutaneous or subcutaneous, and grade the cutaneous MCTs based on the Patnaik and Kiupel classification systems (VET MED LABOR GMBH—Division of IDEXX Laboratories, Leipzig, Germany). All tumors were also stained in the same commercial laboratory with antibodies for the Ki-67 marker and the number of positive cells per 1 cm^2^ grid-area with the highest proliferation activity were recorded. In addition, the KIT expression pattern was described as perimembranous (pattern I), stippled cytoplasmic (pattern II), or diffuse cytoplasmic (pattern III).

### 2.5. Measurement of Biochemical Analytes in the Blood Serum

Blood was collected from the jugular vein on the day of the surgical excision, before any handling or medication. Clot activator tubes were used, in which the whole blood was left to rest for 20 min and then centrifuged for 10 min in 3000 rpm. Serum aliquots were harvested and stored at −80 °C until further analysis. All analyses were conducted in a blinded manner concerning the prognostic factors of the mast cell tumors (MCTs) to eliminate any potential bias in the evaluation of the results.

The paraoxonase-1 (PON-1) activity assay was performed to assess the enzyme’s hydrolytic activity using 4-nitrophenyl acetate as the substrate as previously described [19]. PON-1 catalyzes the hydrolysis of 4-nitrophenyl acetate into 4-nitrophenol, which is measured spectrophotometrically at 405 nm. The increase in absorbance corresponds to the formation of 4-nitrophenol, reflecting PON-1 activity. Enzyme activity is expressed in units per liter (IU/mL), where one unit represents the amount of enzyme that hydrolyzes 1 µmol of 4-nitrophenyl acetate per minute under the assay conditions. An automated clinical biochemistry analyzer (Olympus AU400, Chemistry Analyzer, Beckman Coulter, Brea, CA, USA) was used for measurement.

The activity of butyrylcholinesterase (BChE) was measured using butyrylthiocholine as the substrate. BChE catalyzes the hydrolysis of butyrylthiocholine into thiocholine, which reacts with 5,5′-dithiobis(2-nitrobenzoic acid) (DTNB) to produce a yellow-colored compound, 5-thio-2-nitrobenzoate. The rate of the reaction was monitored spectrophotometrically at 412 nm, with the increase in absorbance being proportional to BChE activity. Results are expressed in micromoles per milliliter per minute (μmol/mL·min), representing the amount of enzyme needed to hydrolyze 1 μmol of butyrylthiocholine per minute under the assay conditions. An automated clinical biochemistry analyzer (Olympus AU400, Chemistry Analyzer, Beckman Coulter, Brea, CA, USA) was used for measurement.

The d-ROMs test, a commercially available assay (Diacron International, Grosseto, Italy) was used to assess oxidative stress by measuring the concentration of reactive oxygen metabolites in the sample according to the previously validated procedure [38]. This test is based on the principle that hydroperoxides, which are generated during oxidative processes, react with a chromogenic substrate (N,N-diethyl-p-phenylenediamine) in the presence of iron ions, forming a colored radical cation. The intensity of the color, measured spectrophotometrically at 505 nm using the Free Carpe Diem analyzer (Diacron International, Grosseto, Italy), is directly proportional to the concentration of ROMs in the sample, reflecting the level of oxidative stress. Results are expressed in Carratelli units (CARR U), where 1 CARR U corresponds to 0.08 mg/dL of hydrogen peroxide.

The total antioxidant capacity was evaluated using both the OXY-adsorbent assay and the CUPRAC method.

The CUPRAC assay involves the reduction in cupric ions (Cu^2^⁺) to cuprous ions (Cu⁺) by antioxidants in the sample using a validated automated assay [39]. In the presence of neocuproine, the reduced Cu⁺ forms a colored complex, which is measured spectrophotometrically at 450 nm. The intensity of the color is directly proportional to the antioxidant capacity. The results are expressed in mmol/L, reflecting the concentration of antioxidants capable of reducing copper ions in the sample. An automated clinical biochemistry analyzer (Olympus AU400, Chemistry Analyzer, Beckman Coulter, Brea, CA, USA) was used for measurement.

The commercially available OXY-adsorbent assay (Diacron International, Grosseto, Italy) is a spectrophotometric method to measure antioxidant capacity in serum samples, previously used in canine samples [40]. This approach involves the oxidation of physiological antioxidants, such as uric acid, glutathione, thiol groups, vitamins, glutathione peroxidase, superoxide dismutase, and catalase, by a high concentration of hypochlorous acid (HClO) in the serum. Following a 10 min incubation period, any remaining HClO reacts with an alkyl-substituted aromatic amine (A-NH2) dissolved in a chromogenic mixture. The reaction oxidizes the amine, generating a colored product that is quantified spectrophotometrically. The intensity of the color measured with the Free Carpe Diem analyzer (Diacron International, Grosseto, Italy) is inversely related to the sample’s antioxidant capacity and directly proportional to the amount of HClO in the sample. The results are expressed as micromoles of HClO consumed per milliliter of sample (μmol HClO/mL).

### 2.6. Statistical Analysis

The dogs were divided into groups based on the histologic location of the neoplasm (cutaneous or subcutaneous). Cutaneous neoplasms were divided into further groups based on the Kiupel (high- and low-grade) and Patnaik (grade 1, 2 or 3) histologic grading. The patients were also grouped based on immunohistochemical staining for Ki67 (>23 or ≤23 positive nuclei per cm^2^ grid) and KIT pattern (I or II–III). ANOVA and independent t-test were used for detecting differences in mean values of the measured variables among different groups with significance level of a = 0.05. All analyses were performed using R Statistical Software (R Core Team 2021).

## 3. Results

### 3.1. Epidemiological Data

A total of 39 dogs with MCT were included in the study, including 24 female and 15 male dogs, with a median age of 9 years (range: 3–14 years). They comprised 15 mixed-breed and 24 purebred dogs, including Pitbulls (*n* = 7), Boxers (*n* = 4), Maltese (*n* = 3) and 1 each of the following breeds: Labrador Retriever, Epagneul Breton, Golden Retriever, French Bulldog, Schnauzer, Cane Corso, Dogo Argentino, Yorkshire Terrier, Shar Pei, and Caucasian Shepherd. These data are summarized in Table 1.

### 3.2. Distribution of Mast Cell Tumors According to Histologic Location, Clinical Staging, Histologic Grading, and Immunohistochemistry

In this study, a total of 33 mast cell tumors (MCTs) were classified according to the Kiupel system, with 25 classified as low-grade and 8 as high-grade. Based on the Patnaik classification, 1 MCT was categorized as grade 1, 25 as grade 2, and 7 as grade 3. Tumor histology also showed that 33 were cutaneous, while 6 were subcutaneous. Regarding metastasis status, 24 MCTs were non-metastatic, while 15 showed evidence of metastasis. Ki67 proliferation index revealed that 12 tumors had more than 23 positive nuclei per 1 cm^2^ grid, and 27 had 23 or fewer. KIT immunohistochemistry showed that 23 tumors exhibited pattern I, 14 pattern II, and 2 pattern III. These findings are summarized in Table 2.

### 3.3. Comparison of the Biochemical Analytes Based on Kiupel Histologic Grading System

**Figure 1:**
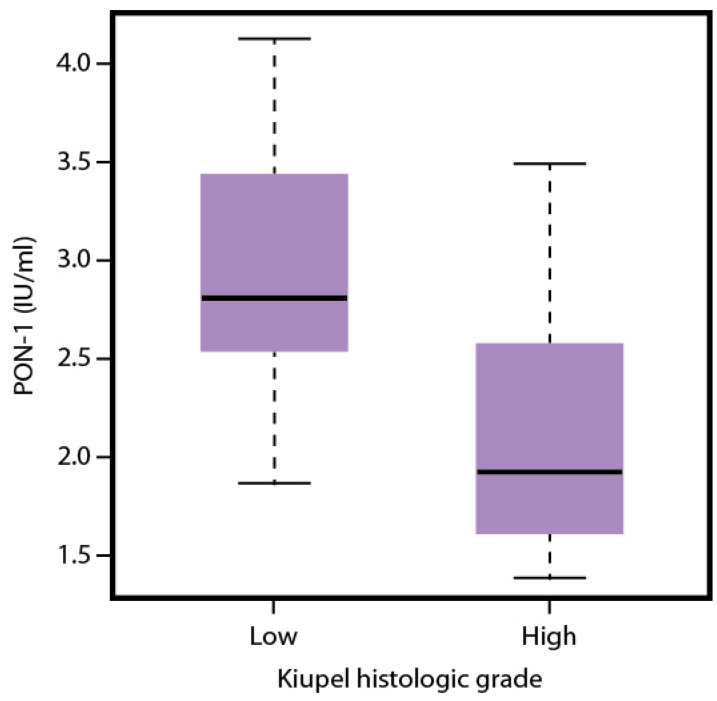
Boxplot illustrating Paraoxonase-1 (PON-1) activity in dogs with cutaneous (*n* = 33) mast cell tumors (MCTs) categorized by Kiupel histologic grading. A total of 25 dogs had low-grade MCTs, and 8 had high-grade MCTs. The mean PON-1 activity was significantly lower in high-grade MCTs compared to low-grade MCTs (*p =* 0.022).

The mean (±SD) values of the measured analytes in dogs with low- and high-grade MCTs, according to the Kiupel classification, are presented in Table 3. The mean values of PON-1 were significantly higher in the low-grade neoplasms (Figure 1). No significant differences were found for the other redox analytes.

### 3.4. Comparison of the Biochemical Analytes Based on Patnaik Histologic Grading System

**Figure 2:**
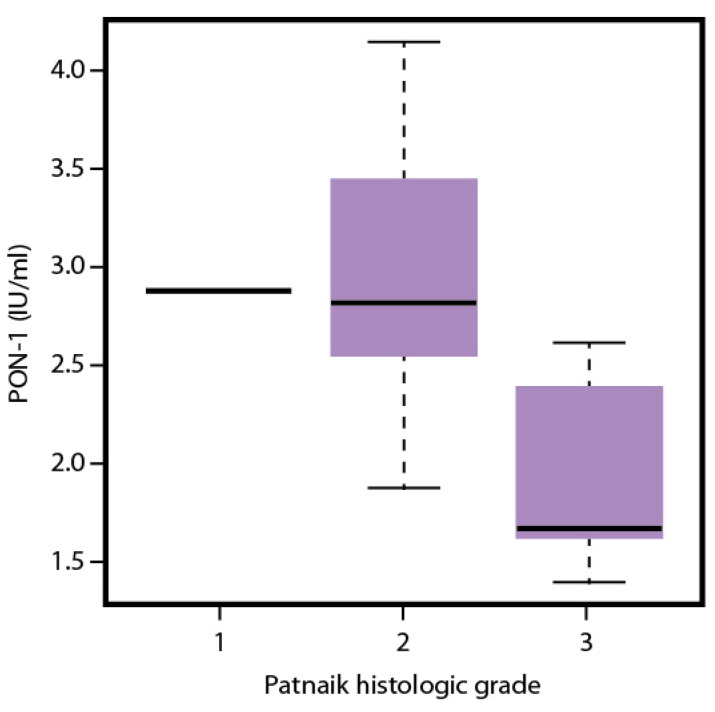
Boxplot illustrating Paraoxonase-1 (PON-1) activity in dogs with cutaneous (*n* = 33) mast cell tumors (MCTs) categorized by Patnaik histologic grading. In total, 1 dog had a grade 1 MCT, 25 had grade 2 MCTs, and 7 had grade 3 MCTs. The mean PON-1 activity was significantly lower in grade 3 MCTs (*p* = 0.003).

The mean (±SD) values of the biochemical analytes in dogs with grades 1, 2, and 3 MCTs, according to Patnaik’s classification, are presented in Table 4. The mean values of PON-1 differed significantly among grades, with lower values measured in dogs belonging to grade 3 (Figure 2). No significant differences were found for the other analytes.

### 3.5. Comparison of the Biochemical Analytes Based on the Histologic Location of MCTs

The mean (±SD) values of the biochemical analytes in dogs with cutaneous (*n* = 33) and subcutaneous (*n* = 6) MCTs are presented in Table 5. Redox analytes did not differ between the two groups.

### 3.6. Comparison of the Biochemical Analytes According to MCTs, Metastatic Status

The mean (±SD) values of the measured analytes with regard to the metastatic status of the tumors are included in Table 6. No significant differences were found in the redox status between dogs with metastatic and with no metastatic MCTs.

### 3.7. Comparison of the Biochemical Analytes in MCTs Evaluated with the Ki67 Proliferation Index

Table 7 presents the mean values of the biochemical analytes in dogs with MCTs evaluated by the Ki67 proliferation index. No significant differences were found between dogs with normal and increased Ki67 labeled cells.

### 3.8. Comparison of the Biochemical Analytes in Dogs with Different KIT Expression Patterns

The mean values of the biochemical analytes in MCTs with pattern I vs. patterns II–III are presented in Table 8. No significant differences were found based on the KIT expression pattern.

## 4. Discussion

This study aimed to investigate analytes related to oxidative status and lipid metabolism in dogs with MCTs and explore their association with well-established prognostic factors of MCTs. Of the assessed panel of markers, only PON-1 activity showed a significant association with MCTs grading, being significantly reduced in dogs with high-grade MCTs.

The finding of decreased PON-1 activity in dogs with high-grade MCTs, according to both the Kiupel and Patnaik grading systems, highlights the potential role of oxidative stress in tumor progression, as well as the potential prognostic role of PON-1 levels in canine MCTs. Importantly, if validated, PON-1 could serve as a clinically relevant biomarker that can be measured in blood prior to surgery, aiding in preoperative risk stratification and treatment planning. PON-1 is an enzyme with known antioxidant properties, and its reduced activity suggests increased oxidative stress in dogs with more aggressive tumors. This result is consistent with findings from other studies, which show that oxidative stress is closely linked to tumor progression, DNA damage, and inflammation [41,42]. Multiple studies have demonstrated that oxidative stress correlates with more aggressive tumor behavior and poorer prognostic outcomes. In canine mammary carcinoma, dogs with tumors exhibited higher oxidative stress levels compared to healthy dogs, with significant reductions observed following surgical treatment [43,44]. Similarly, in cases of multicentric lymphoma, oxidative damage was elevated both before and after chemotherapy, particularly in advanced stages, with increased antioxidant capacity observed in dogs that responded well to treatment [45]. Another study highlighted an imbalance in oxidative and antioxidant markers in dogs with lymphoma, which normalized following clinical remission [46]. Overall, these findings emphasize the role of oxidative stress as a key factor in cancer prognosis.

The medical literature provides extensive evidence on the measurement of blood redox status in various human cancers. Oxidative stress, as measured by 8-hydroxydeoxyguanosine (8-OHdG), has been linked to the development of cancer, while elevated Malondialdehyde (MDA) and reduced vitamin E levels correlate with unfavorable breast cancer outcomes [29,30]. In women with ovarian carcinoma, elevated 8-OHdG concentrations are also associated with a poor prognosis [31]. Additionally, a study on 53 colorectal cancer patients, using d-ROMs to assess oxidative status, identified oxidative stress as a potential independent prognostic marker [47]. This evidence supports the idea that oxidative stress plays a critical role not only in cancer development but also in determining disease outcome.

Interestingly, no association was found between PON-1 concentration and MCT metastasis and IHC markers, despite the fact that they are associated with more advanced grading status. This may underline the fact that the best proxy of MCT behavior is the histologic grade rather than the remainder variables assessed in this study. The lack of correlation between PON-1 and metastasis or IHC markers suggests that oxidative stress as measured by PON-1 may be influenced by tumor biology in a manner independent of these commonly assessed factors. Furthermore, it highlights the complexity of MCT pathophysiology, where not all prognostic indicators may converge on the same mechanistic pathways.

Notably, there was also no association found between the other oxidative and lipid metabolism analytes evaluated in this study (BChE, d-ROMs, CUPRAC, oxy-adsorbent capacity) and tumor grading, metastasis, Ki67 index, and KIT pattern, likely indicating that they are of limited clinical importance as redox status indicators compared to PON-1 in dogs with MCTs. However, the limited sample size may have influenced these results. For instance, the CUPRAC and oxy-adsorbent assays demonstrated variation across histologic grades, but the observed differences did not reach statistical significance (*p* = 0.066 and *p* = 0.077, respectively). This discrepancy may also reflect the inherent differences in the nature of the markers. PON-1 is a relatively stable biomarker, offering insight into cumulative oxidative stress over time, while the other assays provide more transient, momentary snapshots of the oxidative and antioxidant status. Total antioxidant levels can change rapidly with dietary intake or acute stressors, whereas PON-1 activity may reflect a cumulative effect of oxidative stress over time [48]. Moreover, in conditions such as nephropathy and Crohn’s disease, PON-1’s levels have been shown to correlate with the severity of oxidative stress and inflammation, further supporting its role as a long-term biomarker [49,50]. This distinction may explain the stronger association of PON-1 with tumor grading and highlights its potential as a more robust and clinically relevant marker.

Previous reports have suggested that subcutaneous MCTs are generally less aggressive than their cutaneous counterparts [51]. However, the oxidative analytes assessed in this study did not reflect this biological difference, possibly due to the relatively small sample size of subcutaneous tumors.

Several limitations of this study should be acknowledged. First, the relatively small sample size, particularly in certain subgroups, such as subcutaneous MCTs and high-grade tumors, may have limited our ability to detect significant associations across all redox analytes. Larger studies would be required to confirm the findings and to explore the relationship between oxidative status and MCT progression more comprehensively, as well as examine the correlation of oxidative analytes with prognostic factors in subcutaneous MCTs [3,4] and metastatic lymph node grade [52]. Additionally, only a limited range of oxidative markers were assessed in this study. While PON-1 was significantly correlated with tumor grading, other oxidative stress markers such as d-ROMs did not show similar trends, suggesting the need for a broader evaluation of oxidative stress pathways and measurement of more stable analytes. Finally, this preliminary study did not take into account the time to relapse and median survival time of the affected animals. This would be crucial in the attempt to validate PON-1 activity as an independent prognostic factor in canine MCTs. Future studies are ongoing and will account for these variables and employ larger samples to better understand the role of oxidative stress in canine MCTs.

## 5. Conclusions

The results of this study suggest that while PON-1 may be an important marker of oxidative stress in high-grade MCTs, other oxidative and lipid metabolism analytes do not appear to be of clinical importance in tumor progression or prognosis. This highlights the need for a more targeted investigation of specific oxidative pathways that may contribute to tumor behavior in dogs with MCTs. Our findings suggest the potential role of PON-1 as a prognostic biomarker in canine MCTs, since it correlates significantly with histologic grading, the most important established prognostic factor. However, its utility as a prognostic tool requires further validation in larger-scale studies. Future research should address current limitations by including larger populations and longitudinal designs that assess time to relapse and survival time. Expanding the scope of investigation to explore additional oxidative stress markers and their interactions with tumor biology could also uncover novel insights into MCT progression. Further studies with a larger population that will include time to relapse and survival time are ongoing.

## Figures and Tables

**Table 1:** Epidemiological data of the dogs with mast cell tumors included in the study.

Characteristic	Details
Total number of dogs	39
Gender	24 Female (61.5%) 15 Male (38.5%)
Age	Median: 9 years Range: 3–14 years
Breed Distribution	Mixed-breed: 15 dogs (38.5%) Purebred: 24 dogs (61.5%)
Purebred Breakdown	Pitbull: 7 dogs (17.9%) Boxer: 4 dogs (10.3%) Maltese: 3 dogs (7.7%) Other Breeds (1 dog each): Labrador Retriever, Epagneul Breton, Golden Retriever, French Bulldog, Schnauzer, Cane Corso, Dogo Argentino, Yorkshire Terrier, Shar Pei, Caucasian Shepherd (2.6% each)

**Table 2:** Distribution of mast cell tumors (MCTs) by classification, location, metastatic status, Ki67 proliferation index, and KIT staining patterns.

Parameter	Number of Cases	Percentage (%) *
Kiupel Classification		
- Low-grade	25	64.1
- High-grade	8	20.5
- Subcutaneous tumors (grading not applicable)	6	15.4
Patnaik Classification		
- Grade 1	1	2.6
- Grade 2	25	64.1
- Grade 3	7	17.9
- Subcutaneous tumors (grading not applicable)	6	15.4
Tumor histologic location		
- Cutaneous	33	84.6
- Subcutaneous	6	15.4
Metastatic status		
- Non-metastatic	24	61.5
- Metastatic	15	38.5
Ki67 Positive Nuclei per 1 cm^2^ Grid		
- >23	12	30.8
- ≤23	27	69.2
KIT pattern		
- Pattern I	23	59.0
- Pattern II	14	35.9
- Pattern III	2	5.1

* Percentages are based on the total number of dogs (*n* = 39). Kiupel and Patnaik classifications are applied to 33 cutaneous tumors.

**Table 3:** Mean (±SD) values of biochemical analytes in 33 dogs with cutaneous mast cell tumors (MCTs) graded by the 2-tier Kiupel histologic system.

Kiupel Grade	PON-1 (IU/mL)	BCHE (μmol/mL·min)	CUPRAC (mmol/L)	d-ROMs (CARR U)	OXY-Adsorbent (μmol HClO/mL)
Low-grade (*n* = 25)	2.91 ± 0.62	4.83 ± 1.60	0.30 ± 0.05	93.96 ± 19.88	291.98 ± 48.80
High-grade (*n* = 8)	2.16 ± 0.72	4.36 ± 1.67	0.27 ± 0.04	91.38 ± 19.02	252.71 ± 50.22
*p*-value	0.022	0.502	0.066	0.746	0.077

Abbreviations of Table 3: PON-1: Paraoxonase-1, BCHE: Butyrylcholinesterase, CUPRAC: Cupric Reducing Antioxidant Capacity, d-ROMs: Diacron Reactive Oxygen Metabolites, OXY-Adsorbent: Diacron Oxygen Adsorbing Substances Assay.

**Table 4:** Mean (±SD) values of biochemical analytes in 33 dogs with cutaneous mast cell tumors (MCTs) graded by the 3-tier Patnaik histologic system.

Patnaik Grade	PON-1 (IU/mL)	BCHE (μmol/mL·min)	CUPRAC (mmol/L)	d-ROMs (CARR U)	OXY-Adsorbent (μmol HClO/mL)
Grade 1 (*n* = 1)	2.88 ± na	5.60 ± na	0.26 ± na	73.00 ± na	226.50 ± na
Grade 2 (*n* = 25)	2.94 ± 0.63	4.88 ± 1.65	0.30 ± 0.05	93.64 ± 20.30	291.42 ± 49.66
Grade 3 (*n* = 7)	1.96 ± 0.50	4.00 ± 1.42	0.27 ± 0.05	95.14 ± 17.01	258.46 ± 51.32
*p*-value	0.003	0.387	0.172	0.574	0.177

Abbreviations of Table 4: PON-1: Paraoxonase-1, BCHE: Butyrylcholinesterase, CUPRAC: Cupric Reducing Antioxidant Capacity, d-ROMs: Diacron Reactive Oxygen Metabolites, OXY-Adsorbent: Diacron Oxygen Adsorbing Substances Assay, na: Not Applicable.

**Table 5:** Mean (±SD) values of biochemical analytes in dogs with cutaneous or subcutaneous mast cell tumors.

Histologic Location	PON-1 (IU/mL)	BCHE (μmol/mL·min)	CUPRAC (mmol/L)	d-ROMs (CARR U)	OXY-Adsorbent (μmol HClO/mL)
Cutaneous (*n* = 33)	2.73 ± 0.71	4.72 ± 1.60	0.30 ± 0.05	93.33 ± 19.41	282.46 ± 51.28
Subcutaneous (*n* = 6)	2.18 ± 0.57	3.60 ± 1.19	0.28 ± 0.07	96.17 ± 18.02	266.75 ± 35.23
*p*-value	0.070	0.079	0.668	0.736	0.377

Abbreviations of Table 5: PON-1: Paraoxonase-1, BCHE: Butyrylcholinesterase, CUPRAC: Cupric Reducing Antioxidant Capacity, d-ROMs: Diacron Reactive Oxygen Metabolites, OXY-Adsorbent: Diacron Oxygen Adsorbing Substances Assay.

**Table 6:** Mean (±SD) values of biochemical analytes in dogs with metastatic and not metastatic cutaneous/subcutaneous mast cell tumors.

Metastatic Status	PON-1 (IU/mL)	BCHE (μmol/mL·min)	CUPRAC (mmol/L)	d-ROMs (CARR U)	OXY-Adsorbent (μmol HClO/mL)
Metastatic (*n* = 15)	2.74 ± 0.61	4.73 ± 1.35	0.29 ± 0.05	92.25 ± 21.06	284.06 ± 47.04
Non-metastatic (*n* = 24)	2.49 ± 0.85	4.24 ± 1.92	0.29 ± 0.06	96.20 ± 15.55	273.61 ± 53.26
*p*-value	0.318	0.393	0.887	0.506	0.538

Abbreviations of Table 6: PON-1: Paraoxonase-1, BCHE: Butyrylcholinesterase, CUPRAC: Cupric Reducing Antioxidant Capacity, d-ROMs: Diacron Reactive Oxygen Metabolites, OXY-Adsorbent: Diacron Oxygen Adsorbing Substances Assay.

**Table 7:** Mean (±SD) values of biochemical analytes in dogs with cutaneous/subcutaneous mast cell tumors (MCTs) evaluated by the Ki67 index.

No of Ki67 Positive Nuclei per cm^2^	PON-1 (IU/mL)	BCHE (μmol/mL·min)	CUPRAC (mmol/L)	d-ROMs (CARR U)	OXY-Adsorbent (μmol HClO/mL)
>23 (*n* = 12)	2.59 ± 0.70	4.28 ± 1.08	0.30 ± 0.06	89.41 ± 10.49	292.81 ± 48.01
<=23 (*n* = 27)	2.39 ± 0.77	4.18 ± 1.67	0.27 ± 0.04	90.54 ± 19.71	264.52 ± 50.61
*p*-value	0.464	0.856	0.887	0.854	0.133

Abbreviations of Table 7: PON-1: Paraoxonase-1, BCHE: Butyrylcholinesterase, CUPRAC: Cupric Reducing Antioxidant Capacity, d-ROMs: Diacron Reactive Oxygen Metabolites, OXY-Adsorbent: Diacron Oxygen Adsorbing Substances Assay.

**Table 8:** Mean (±SD) values of biochemical analytes in dogs with cutaneous/subcutaneous mast cell tumors (MCTs) evaluated by the KIT expression patterns.

KIT Pattern	PON-1 (IU/mL)	BCHE (μmol/mL·min)	CUPRAC (mmol/L)	d-ROMs (CARR U)	OXY-Adsorbent (μmol HClO/mL)
I (*n* = 23)	2.69 ± 0.82	4.14 ± 1.36	0.30 ± 0.07	88.7 ± 10.29	300.81 ± 50.37
II–III (*n* = 16)	2.41 ± 0.67	4.29 ± 1.37	0.28 ± 0.05	90.5 ± 16.95	270.43 ± 48.41
*p*-value	0.364	0.780	0.364	0.721	0.133

Abbreviations of Table 8: PON-1: Paraoxonase-1, BCHE: Butyrylcholinesterase, CUPRAC: Cupric Reducing Antioxidant Capacity, d-ROMs: Diacron Reactive Oxygen Metabolites, OXY-Adsorbent: Diacron Oxygen Adsorbing Substances Assay.

## Data Availability

The data presented in this study are available on request from the corresponding author due to ethical restrictions.

## References

[1] Bostock D.E. (1986). Neoplasms of the Skin and Subcutaneous Tissues in Dogs and Cats. Br. Vet. J..

[2] Kiupel M., Webster J.D., Bailey K.L., Best S., DeLay J., Detrisac C.J., Fitzgerald S.D., Gamble D., Ginn P.E., Goldschmidt M.H. (2011). Proposal of a 2-tier histologic grading system for canine cutaneous mast cell tumors to more accurately predict biological behavior. Vet. Pathol..

[3] Thompson J.J., Pearl D.L., Yager J.A., Best S.J., Coomber B.L., Foster R.A. (2011). Canine subcutaneous mast cell tumor: Characterization and prognostic indices. Vet. Pathol..

[4] Thompson J.J., Yager J.A., Best S.J., Pearl D.L., Coomber B.L., Torres R.N., Kiupel M., Foster R.A. (2011). Canine subcutaneous mast cell tumors: Cellular proliferation and KIT expression as prognostic indices. Vet. Pathol..

[5] Romansik E.M., Reilly C.M., Kass P.H., Moore P.F., London C.A. (2007). Mitotic index is predictive for survival for canine cutaneous mast cell tumors. Vet. Pathol..

[6] Kiupel M., Webster J.D., Kaneene J.B., Miller R., Yuzbasiyan-Gurkan V. (2004). The use of KIT and tryptase expression patterns as prognostic tools for canine cutaneous mast cell tumors. Vet. Pathol..

[7] Scase T.J., Edwards D., Miller J., Henley W., Smith K., Blunden A., Murphy S. (2006). Canine mast cell tumors: Correlation of apoptosis and proliferation markers with prognosis. J. Vet. Intern. Med..

[8] Ozaki K., Yamagami T., Nomura K., Narama I. (2007). Prognostic Significance of Surgical Margin, Ki-67 and Cyclin D1 Protein Expression in Grade II Canine Cutaneous Mast Cell Tumor. J. Vet. Med. Sci..

[9] Webster J.D., Yuzbasiyan-Gurkan V., Miller R.A., Kaneene J.B., Kiupel M. (2007). Cellular proliferation in canine cutaneous mast cell tumors: Associations with c-KIT and its role in prognostication. Vet. Pathol..

[10] Thamm D.H., Vail D.M., Withrow S.J., Vail D.M. (2007). Mast cell tumours. Small Animal Clinical Oncology.

[11] Horta R.S., LaValle G.E., Monteiro L.N., Souza M.C.C., Cassali G.D., Araújo R.B. (2018). Assessment of Canine Mast Cell Tumor Mortality Risk Based on Clinical, Histologic, Immunohistochemical, and Molecular Features. Vet. Pathol..

[12] Sies H., Berndt C., Jones D.P. (2017). Oxidative Stress. Annu. Rev. Biochem..

[13] Harwell B. (2007). Biochemistry of oxidative stress. Biochem. Soc. Trans..

[14] Kilk K., Meitern R., Härmson O., Soomets U., Hõrak P. (2014). Assessment of oxidative stress in serum by d-ROMs test. Free Radic. Res..

[15] Tsamesidis I., Egwu C.O., Samara D., Vogiatzi D., Lettas A., Lymperaki E. (2022). Effects of Greek Honey and Propolis on Oxidative Stress and Biochemical Analytes in Regular Blood Donors. J. Xenobiot..

[16] Takaki J. (2013). Associations of Job Stress Indicators with Oxidative Biomarkers in Japanese Men and Women. Int. J. Environ. Res. Public Health.

[17] Katerji M., Filippova M., Duerksen-Hughes P. (2019). Approaches and Methods to Measure Oxidative Stress in Clinical Samples: Research Applications in the Cancer Field. Oxid. Med. Cell Longev..

[18] Oberacker T., Kraft L., Schanz M., Latus J., Schricker S. (2023). The Importance of Thioredoxin-1 in Health and Disease. Antioxidants.

[19] Ceron J.J., Tecles F., Tvarijonaviciute A. (2014). Serum paraoxonase 1 (PON1) measurement: An update. BMC Vet. Res..

[20] Nuhu F., Gordon A., Sturmey R., Seymour A.-M., Bhandari S. (2020). Measurement of Glutathione as a Tool for Oxidative Stress Studies by High Performance Liquid Chromatography. Molecules.

[21] Ford E.S., Schleicher R.L., Mokdad A.H., Ajani U.A., Liu S. (2006). Distribution of serum concentrations of alpha-tocopherol and gamma-tocopherol in the US population. Am. J. Clin. Nutr..

[22] Munteanu I.G., Apetrei C. (2021). Analytical Methods Used in Determining Antioxidant Activity: A Review. Int. J. Mol. Sci..

[23] Durrington P.N., Bashir B., Soran H. (2023). Paraoxonase 1 and atherosclerosis. Front. Cardiovasc. Med..

[24] Soran H., Schofield J.D., Durrington P.N. (2015). Antioxidant properties of HDL. Front. Pharmacol..

[25] Medina-Díaz I.M., Ponce-Ruíz N., Rojas-García A.E., Zambrano-Zargoza J.F., Bernal-Hernández Y.Y., González-Arias C.A., Barrón-Vivanco B.S., Herrera-Moreno J.F. (2022). The Relationship between Cancer and Paraoxonase 1. Antioxidants.

[26] Gensthaler L., Jomrich G., Brugger J., Kollmann D., Paireder M., Bologheanu M., Horn A., Riegler F.M., Asari R., Schoppmann S.F. (2023). Preoperative BChE serves as a prognostic marker in patients with resectable AEG after neoadjuvant chemotherapy. Langenbecks Arch. Surg..

[27] Lampón N., Hermida-Cadahia E.F., Riveiro A., Tutor J.C. (2012). Association between butyrylcholinesterase activity and low-grade systemic inflammation. Ann. Hepatol..

[28] Liu J., Tian T., Liu X., Cui Z. (2022). BCHE as a Prognostic Biomarker in Endometrial Cancer and Its Correlation with Immunity. J. Immunol. Res..

[29] Loft S., Olsen A., Møller P., Poulsen H.E., Tjønneland A. (2013). Association between 8-oxo-7,8-dihydro-2′-deoxyguanosine excretion and risk of postmenopausal breast cancer: Nested case-control study. Cancer Epidemiol. Biomark. Prev..

[30] Saintot M., Mathieu-Daude H., Astre C., Grenier J., Simony-Lafontaine J., Gerber M. (2002). Oxidant-antioxidant status in relation to survival among breast cancer patients. Int. J. Cancer.

[31] Pylväs M., Puistola U., Laatio L., Kauppila S., Karihtala P. (2011). Elevated serum 8-OHdG is associated with poor prognosis in epithelial ovarian cancer. Anticancer Res..

[32] Rubio C.P., Martinez-Subiela S., Tvarijonaviciute A., Hernández-Ruiz J., Pardo-Marin L., Segarra S., Ceron J.J. (2016). Changes in serum biomarkers of oxidative stress after treatment for canine leishmaniosis in sick dogs. Comp. Immunol. Microbiol. Infect. Dis..

[33] Rubio C.P., Martínez-Subiela S., Hernández-Ruiz J., Tvarijonaviciute A., Cerón J.J., Allenspach K. (2017). Serum biomarkers of oxidative stress in dogs with idiopathic inflammatory bowel disease. Vet. J..

[34] Cucchi A., Ramoni R., Basini G., Bussolati S., Quintavalla F. (2020). Oxidant–Antioxidant Status in Canine Multicentric Lymphoma and Primary Cutaneous Mastocytoma. Processes.

[35] Finotello R., Pasquini A., Meucci V., Lippi I., Rota A., Guidi G., Marchetti V. (2014). Redox status evaluation in dogs affected by mast cell tumour. Vet. Comp. Oncol..

[36] Book A.P., Fidel J., Wills T., Bryan J., Sellon R., Mattoon J. (2011). Correlation of ultrasound findings, liver and spleen cytology, and prognosis in the clinical staging of high metastatic risk canine mast cell tumors. Vet. Radiol. Ultrasound.

[37] Warland J., Amores-Fuster I., Newbury W., Brearley M., Dobson J.M. (2012). The utility of staging in canine mast cell tumours. Vet. Comp. Oncol..

[38] Pasquini A., Luchetti E., Marchetti V., Cardini G., Iorio E.L. (2008). Analytical performances of d-ROMs test and BAP test in canine plasma. Definition of the normal range in healthy Labrador dogs. Vet. Res. Commun..

[39] Rubio C.P., Tvarijonaviciute A., Martinez-Subiela S., Hernández-Ruiz J., Cerón J.J. (2016). Validation of an automated assay for the measurement of cupric reducing antioxidant capacity in serum of dogs. BMC Vet. Res..

[40] Candellone A., Girolami F., Badino P., Jarriyawattanachaikul W., Odore R. (2022). Changes in the Oxidative Stress Status of Dogs Affected by Acute Enteropathies. Vet. Sci..

[41] Nemec Svete A., Verk B., Čebulj-Kadunc N., Salobir J., Rezar V., Domanjko Petrič A. (2021). Inflammation and its association with oxidative stress in dogs with heart failure. BMC Vet. Res..

[42] Poetsch A.R. (2020). The genomics of oxidative DNA damage, repair, and resulting mutagenesis. Comput. Struct. Biotechnol. J..

[43] Machado V.S., Crivellenti L.Z., Bottari N.B., Tonin A.A., Pelinson L.P., Borin-Crivellenti S., Santana A.E., Torbitz V.D., Moresco R.N., Duarte T. (2015). Oxidative stress and inflammatory response biomarkers in dogs with mammary carcinoma. Pathol. Res. Pract..

[44] Russo C., Simonelli S.M., Luz M.B., Carrera A.C., Moreno I.F., Bracarense A.P.F.R.L. (2021). Oxidative stress in female dogs with mammary neoplasms. Pesq. Vet. Bras..

[45] Bottari N.B., Munhoz T.D., Torbitz V.D., Tonin A.A., Anai L.A., Semolin L.M., Jark P.C., Bollick Y.S., Moresco R.N., França R.T. (2015). Oxidative stress in dogs with multicentric lymphoma: Effect of chemotherapy on oxidative and antioxidant biomarkers. Redox Rep..

[46] Winter J.L., Barber L.G., Freeman L., Griessmayr P.C., Milbury P.E., Blumberg J.B. (2009). Antioxidant status and biomarkers of oxidative stress in dogs with lymphoma. J. Vet. Intern. Med..

[47] Sugimoto K., Sakamoto K., Kawai M., Kawano S., Munakata S., Ishiyama S., Takahashi M., Kojima Y., Tomiki Y. (2019). Serum oxidative stress is an independent prognostic marker in colorectal cancer. Transl. Cancer Res..

[48] Roxo D.F., Arcaro C.A., Gutierres V.O., Costa M.C., Oliveira J.O., Lima T.F.O., Assis R.P., Brunetti I.L., Baviera A.M. (2019). Curcumin combined with metformin decreases glycemia and dyslipidemia, and increases paraoxonase activity in diabetic rats. Diabetol. Metab. Syndr..

[49] Szczeklik K., Mach T., Cibor D., Owczarek D., Sapa J., Papież M., Pytko-Polończyk J., Krzyściak W. (2018). Correlation of Paraoxonase-1 with the Severity of Crohn’s Disease. Molecules.

[50] Hashemi M., Sadeghi-Bojd S., Raeisi M., Moazeni-Roodi A. (2013). Evaluation of paraoxonase activity in children with nephrotic syndrome. Nephrourol. Mon..

[51] de Nardi A.B., dos Santos Horta R., Fonseca-Alves C.E., de Paiva F.N., Linhares L.C.M., Firmo B.F., Ruiz Sueiro F.A., de Oliveira K.D., Lourenço S.V., De Francisco Strefezzi R. (2022). Diagnosis, Prognosis and Treatment of Canine Cutaneous and Subcutaneous Mast Cell Tumors. Cells.

[52] Weishaar K.M., Thamm D.H., Worley D.R., Kamstock D.A. (2014). Correlation of nodal mast cells with clinical outcome in dogs with mast cell tumour and a proposed classification system for the evaluation of node metastasis. J. Comp. Pathol..

